# Competence of omeprazole and chitosan nanoparticles as promising approaches for enhancing vegetative growth and leaf anatomical structure of plum under low nitrogen conditions

**DOI:** 10.1186/s12870-025-07852-5

**Published:** 2025-12-13

**Authors:** Mohamed A. Abd-Elfattah, Bahaaaldin M. Mamdouh, Zeinab K. Taha, Ne’ma M. Zaid

**Affiliations:** 1 Pomology Department, Faculty of Agriculture, Cairo University, Giza, 12613 Egypt; 2 Agricultural Botany Department, Faculty of Agriculture, Cairo University, Giza, 12613 Egypt

**Keywords:** Omeprazole, Chitosan nanoparticles, Nitrogen fertilizers, Plum, Solar Eclipse, Suplum 41, Vegetative growth, Leaf anatomy

## Abstract

**Background:**

Reducing the requirements of chemical fertilizers has emerged as a critical target with economic and environmental concerns in agricultural sector. Consequently, there is a growing need for research focused on developing sustainable approaches to enhance fertilizer use efficiency. Omeprazole (OMZ) and chitosan nanoparticles (ChNPs) have shown promising results and are considered as growth promoters. However, to the best of our knowledge there are no available data about the application of OMZ on fruit trees.

**Aim:**

Decreasing utilization rate of N chemical fertilizers while maintaining optimal growth of plum plants (Solar Eclipse and Suplum 41 cvs.) as changes related to vegetative growth and leaf anatomy by application of OMZ and ChNPs had been investigated under low nitrogen conditions.

**Methods:**

Plum grafted seedlings of each cultivar are divided to four groups: (a) Plants received 10 mM NO_3_^−^, (b) Plants received 2 mM NO_3_^−^, (c) Plants received 2 mM NO_3_^−^ loaded on ChNPs, and (d) Plants received OMZ + 2 mM NO_3_^−^. ChNPs were synthesized using the ionotropic gelation and characterized by transmission electron microscope and zetasizer. Vegetative growth parameters including plant height, stem diameter, number of leaves per plant, and leaf area were measured. Photosynthetic pigments and total N content in leaves of plum plants were analyzed. Leaf anatomy was performed to identify leaf structural changes in response to different treatments. One-way analysis of variance was performed for statistical analysis and significant differences (α ≤ 0.05) were determined using Duncan’s multiple range test.

**Results:**

The obtained results revealed that ChNPs and OMZ enhanced vegetative growth and leaf structure compared to untreated plants, even when receiving low nitrogen (2mM NO_3_^−^) or high (10 mM NO_3_^−^). Plum plants treated with OMZ exhibited improvement in growth and leaf structure higher than ChNPs under low nitrogen conditions.

**Conclusion:**

OMZ and ChNPs could be a cost-effective way to save amounts of chemical fertilizers added to soil and increase nutrient use efficiency.

## Introduction

Cultivated plums are mainly derived from two species (European plum and Japanese plum) and belong to the genus *Prunus* within the flowering plant family Rosaceae, which contains all the stone fruits [1, 2]. Plum cultivation in Egypt has expanded due to the release of new plum cultivars characterized by low chilling requirements (adapted to Egyptian climate conditions) and self-compatibility with high-quality fruits. Plum production in Egypt has increased in the last decade to reach about 44,424 tons in 2022 with ~ 60% increase in cultivated area and production compared to statistics of 2020 [3].

Plants required large amounts of nitrogen (N) due to crucial role in protein synthesis and energy metabolism which affect plant growth and metabolism [4]. Nitrogen fertilizers represent a significant cost of production for the grower [5]. Only 30–40% of N applied to the soil is utilized by plants since the N compounds in the form of NO_3_^−^ and NH_4_^+^ are highly mobile in the soil with excessive loss through denitrification, leaching, and volatilization [6–8]. In recent decades, reducing the requirements for N chemical fertilizers has emerged as a critical target that could be achieved by increasing nitrogen use efficiency (NUE) [5, 9]. Maximizing NUE in plants relies on understanding the mechanisms related to N (Nitrate) uptake, assimilation and translocation efficiency [10].

Nano-fertilizers as the new generation of fertilizers like biopolymer-mineral nanocomposites are characterized by an eco-friendly nature, higher stability and biodegradability, high Nutrient uptake efficiency, control of nutrient release, low production cost and less harmful in agricultural practices [11, 12]. Nano-Chitosan-fertilizers are expected to contribute in magnifying the agricultural sector and dealing with the global food crisis [13]. Chitosan is a non-toxic polymer with a high absorbent nature and could supply plants with sustained release of fertilizers and decrease the excessive loss of nutrients as a coating material and obtain high productivity in agriculture [14, 15]. Besides being a nanocarrier, ChNPs can also provide plants with macronutrients and micronutrients through their functional groups [16]. These nano-fertilizers additionally prove to have little to no adverse effects on plant systems [17].

Omeprazole (OMZ) is a member of benzimidazoles in mammals and acts as a proton pump inhibitor [18]. However, it has an influence on other physiological activities in different plant species [19]. OMZ is able to increase the roots uptake of nitrate and increase its concentration in leaves which can favor the synthesis of N-containing compounds involved in many physiological processes [20]. It has been shown to act as a plant growth regulator that modulates natural hormone-signaling networks [21]. Several studies have highlighted a beneficial role of omeprazole in many plant species. OMZ was found to stimulate root growth, improve vegetative growth and enhance photosynthesis in tomato plants [21, 22]. It was proven in lettuce and basil plants that application of OMZ at micromolar concentrations enhanced NO_3_^−^ and K^+^ uptake and their translocation into the shoots [20, 23, 24]. Moreover, OMZ has the ability to increase nutrient use efficiency in plants under normal and stress conditions [20, 25]. Furthermore, it induced changes at the enzyme, metabolic, and gene expression levels which allowed for an increase in N assimilation in maize seedlings [19]. Besides, it increased leaf chlorophyll content, photosynthetic rate, and gas exchange in peppermint plants [26]. Also, foliar application of OMZ (1 µM) was found to increase soluble amino acids and NUE which reflected on the growth and yield of green bean plants [27].

Consequently, Researchers have developed macronutrient nano-fertilizers for field-grown plants. Also, there is evidence of increased NUE with the use of OMZ in some crops. However, to the best of our knowledge there are no available data about the application of OMZ on fruit trees. Moreover, the utilization of ChNPs and their effect on plum grafted seedlings has remained unexplored until now. Additionally, none of the previous studies had addressed the effects of OMZ on leaf anatomy. Therefore, this study is based on the hypothesis that the application of ChNPs and OMZ may reduce the need for N chemical fertilizers while maintaining optimal growth of plum plants. To verify this hypothesis, the objectives of the present study were to evaluate two different approaches through the application of ChNPs-based N fertilizer and OMZ with measuring their efficacy in enhancing vegetative growth and improving leaf anatomical features of plum cultivars (Solar Eclipse and Suplum 41).

## Materials and methods

### Greenhouse conditions

The experiment was conducted for three months in a shade-net greenhouse situated at the Faculty of Agriculture, Cairo university located at Giza, Egypt (30°01’04.6"N 31°12’32.2"E). Temperature was ranged 28 to 35 °C and the relative humidity ranged between 35% and 60% during the growing cycle.

### Plant material

Plum (*Prunus salicina* L.) was tested as a model of woody fruit trees. Plum cultivars (Solar Eclipse and Suplum 41) were grafted on Mariana rootstock. Uniform and healthy grafted plants (1 Year old) were selected and put in black polyethylene bags filled with sandy soil (20 Kg). Planting bags were disposed on bricks in single rows.

### Nutrient solution management

The nutrient solution had the following composition: 1.4 mM P, 6.0 mM K, 4.5 mM Ca and 2.0 mM Mg while N was added in NO_3_^−^ form and its concentration was differed according to treatment. The nutrient solutions for all treatments were pumped from independent plastic tanks (100 L) and delivered to the plants through a drip irrigation system with one dripper per plant (4 L h ^− 1^). Micronutrients were provided three times in sufficient dose during experiment period through foliar spray.

### Description of treatments

Plum plants of each cultivar were divided into four treatments with 9 plants per treatment as following: (1) high nitrogen (10 mM NO_3_^−^), (2) Low nitrogen (2mM NO_3_^−^), (3) Low nitrogen (2mM NO_3_^−^) loaded on ChNPs and (4) Low nitrogen (2mM NO_3_^−^) + OMZ. Nitrate concentrations were selected to facilitate the observation of differences in growth and morphological characteristics between plum plants received low or high concentration. ChNPs-NO_3_ was applied once per week till the end of experiment. OMZ was applied at final concentration of 5 µM as substrate drench treatment during the growing cycle at weekly intervals at a uniform rate of 1 L per plant. Concentration of OMZ (5 µM) was chosen based on ranges used in previous experiments in which indicated that OMZ acts with a hormone-like behavior with growth stimulation between 1 and 10 µM [21, 24], while higher concentrations (50–100 µM) had either a reduction effect or an inhibitory effect [19].

### Preparation of chitosan nanoparticles-based nitrogen fertilizer

ChNPs-NO_3_ were prepared according to method described by Vaezifar et al., [28]. Chitosan - Ch (LOBA Chemie PVT. LTD., Mumbai, India) aqueous solution (0.1% w/v) was prepared by dissolving in acetic acid solution (1% v/v) (Merck, Darmstadt, Germany) at ambient temperature. The pH was adjusted to 4.8 with sodium hydroxide pellets (Merck, Darmstadt, Germany) solution (0.5 M) as final pH reached to 5.5 after preparation. Subsequently, sodium tripolyphosphate - STPP (Oxford, Maharashtra, India) as a cross-linking agent was dissolved in deionized water to a final concentration of 1 mg/mL. After 30 min of vigorous magnetic stirring, the STPP solution was added dropwise (1 mL/min) to the chitosan solution. ChNPs-based nitrogen fertilizer was prepared by loading nitrogen (N) into ChNPs by dissolving calcium nitrate (LOBA Chemie PVT. LTD., Mumbai, India) into nanoparticles solution under homogenizing for 30 min in the presence of Tween 80 at 25 °C [29].

### Characterization of chitosan nanoparticles

#### Morphological characterization of nanoparticles

The morphology including size and shape of the nanoparticles was determined using a transmission electron microscope (JEOL JEM-1400, Peabody, MA, USA) at the Cairo University Research Park (CURP). Diluted ChNPs solution was ultra-sonicated for 5 min to reduce the particle aggregation. Three drops from the sonicated solution were deposited on a carbon-coated copper grid and left to dry at room temperature. HR-TEM images of the ChNPs were captured from multiple fields of view (10 fields with measuring at least 5–10 particles per each field) for morphological evaluation and verify sample homogeneity. The size was obtained by measuring the diameter of the particles present in the TEM image.

#### Size measurements of nanoparticles

Size distribution and polydispersity (PDI) index of ChNPs were determined by dynamic light scattering (DLS) using a Zetasizer (Nano ZS–ZEN3600, Malvern Panalytical Ltd., Malvern, Worcestershire, United Kingdom). Deionized water was used as a reference for dispersing medium with viscosity of 0.89 cP and refractive index of 1.33. Sample was diluted in deionized water (1:10) followed by sonication for 15 min at room temperature. The testing was performed after sonication with scattered light detected at a 90◦ angle and a temperature of 25 °C including three repeated measurements per sample.

### Growth parameters

Plant height (cm) was determined using measuring tape, shoot diameter (mm) was measured using a digital caliper and number of leaves per plant was counted for each plant at the end of experiment (after 10 weeks of treatment).

### Leaf characteristics

Leaf length and width were measured using a ruler. Leaf area (cm^2^) was calculated according to Sabouri et al. [30]., using the following equation:

$$\:\mathrm{L}\mathrm{e}\mathrm{a}\mathrm{f}\:\mathrm{a}\mathrm{r}\mathrm{e}\mathrm{a}\:\left({\mathrm{c}\mathrm{m}}^{2}\right)=\:0.007+0.687\:\left(\mathrm{L}\mathrm{e}\mathrm{a}\mathrm{f}\:\mathrm{l}\mathrm{e}\mathrm{n}\mathrm{g}\mathrm{t}\mathrm{h}\:\times\:\:\mathrm{L}\mathrm{e}\mathrm{a}\mathrm{f}\:\mathrm{w}\mathrm{i}\mathrm{d}\mathrm{t}\mathrm{h}\right)\:\:\:\:\:\:\:\:\left(1\right)$$

Total leaf area (m^2^/plant) was calculated using the following equation:

$$\:\mathrm{T}\mathrm{o}\mathrm{t}\mathrm{a}\mathrm{l}\:\mathrm{L}\mathrm{e}\mathrm{a}\mathrm{f}\:\mathrm{a}\mathrm{r}\mathrm{e}\mathrm{a}\:\left({\mathrm{m}}^{2}\right)=\:\mathrm{L}\mathrm{e}\mathrm{a}\mathrm{f}\:\mathrm{a}\mathrm{r}\mathrm{e}\mathrm{a}\:\times\:\mathrm{N}\mathrm{o}.\:\mathrm{o}\mathrm{f}\:\mathrm{L}\mathrm{e}\mathrm{a}\mathrm{v}\mathrm{e}\mathrm{s}\:\mathrm{p}\mathrm{e}\mathrm{r}\:\mathrm{p}\mathrm{l}\mathrm{a}\mathrm{n}\mathrm{t}\:\:\:\:\:\:\:\:\:\:\:\:\:\:\left(2\right)$$

### Photosynthetic pigments

Leaf samples (0.25 g) were homogenized in 20 mL of 80% (v/v) acetone (LOBA Chemie PVT. LTD., Mumbai, India). The samples were sealed and allowed to stand in the dark for 48 h. The absorbance of the samples was measured at wavelengths of 646, and 663 nm using a UV-visible spectrophotometer (UNICO S2100, Cole Parmer Instruments, Illinois, USA). The pigment concentrations were calculated according to Lichtenthaler [31] and expressed as mg/g FW using the following formulas:

$$\:{\mathrm{C}\mathrm{h}\mathrm{l}}_{\mathrm{a}}\:(\mathrm{m}\mathrm{g}/\mathrm{g}\:\mathrm{F}\mathrm{W})=\frac{12.25\:{\mathrm{A}}_{663}-2.79\:{\mathrm{A}}_{646}\times\:\mathrm{V}}{\mathrm{W}\times\:1000}\:\:\:\:\:\:\:(3)\:\:\:$$

$$\:{\mathrm{C}\mathrm{h}\mathrm{l}}_{\mathrm{b}\:}(\mathrm{m}\mathrm{g}/\mathrm{g}\:\mathrm{F}\mathrm{W})=\frac{21.50\:{\mathrm{A}}_{646}-5.10\:{\mathrm{A}}_{663}\times\:\mathrm{V}}{\mathrm{W}\times\:1000}\:\:\:\:\:\:\:(4)\:\:$$

Where: A_663_ = absorbance readings at 663 wavelengths, A_646_ = absorbance readings at 646 wavelengths, V = Volume of the acetone solution (mL), W = Weight of leaf sample (g).

On the other hand, the leaf chlorophyll index (SPAD) was measured by using a portable chlorophyll meter (Minolta SPAD-502, Minolta corporation Ltd., Osaka, Japan) as a nondestructive method used for estimating total chlorophyll content in plant leaves. SPAD measurements were performed between 7:00 and 9:00 am on 4th week of the experiment period. Five SPAD readings were taken around the edges of fully expanded youngest leaf of each plant and averaged using the internal function of the chlorophyll meter [32].

### Total nitrogen (%)

Total nitrogen was determined based on Kjeldahl method [33]. Leaf powdered sample (1 g) was digested by boiling with 10 mL of concentrated H_2_SO_4_ (ADWIC pharmaceutical chemicals co., Cairo, Egypt) and a catalyst in a digestion tube until the mixture was clear. The digest was allowed to cool, filtered and then processed for distillation. The tube containing the digested sample was placed in the apparatus and the automatic distillation process was started. The sample was made strongly alkaline through addition of sodium hydroxide (Merck, Darmstadt, Germany) solution for fully conversion of the ammonium ions (NH_4_^+^) into ammonia (NH_3_). The released ammonia was steam-distilled into a collector filled with boric acid (LOBA Chemie PVT. LTD., Mumbai, India) solution. When distillation was finished, back titration with 0.1 M HCl (VWR International LLC, Pennsylvania, USA) started to neutralize the ammonium borate complex. Blank sample was prepared with all reagents alone without leaf sample. Nitrogen percentage was calculated as follows:

$$\:Total\:\mathrm{N}\:\left(\%\right)=\frac{{(\mathrm{V}}_{\mathrm{H}\mathrm{C}\mathrm{L}\:\mathrm{s}\mathrm{a}\mathrm{m}\mathrm{p}\mathrm{l}\mathrm{e}}-\:{\mathrm{V}}_{\mathrm{H}\mathrm{C}\mathrm{L}\:\mathrm{b}\mathrm{l}\mathrm{a}\mathrm{n}\mathrm{k}})\:\times\:{\mathrm{C}}_{\mathrm{H}\mathrm{C}\mathrm{l}}\:\times\:{\mathrm{M}}_{\mathrm{N}}}{{\mathrm{W}}_{\mathrm{s}\mathrm{a}\mathrm{m}\mathrm{p}\mathrm{l}\mathrm{e}}}\:\times\:100\:\:\:\:\:\:\left(5\right)$$

Where: V_HCl sample_ = Volume of HCl used in sample titration (mL), V_HCl blank_ = Volume of HCl used in blank titration (mL), C_HCl_ = Concentration of HCl (0.1 M), M_N_ = Molar mass of nitrogen (14.007 g/mol), W_sample_ = Weight of leaf samples (g).

### Anatomical studies

**Fig. 1:**
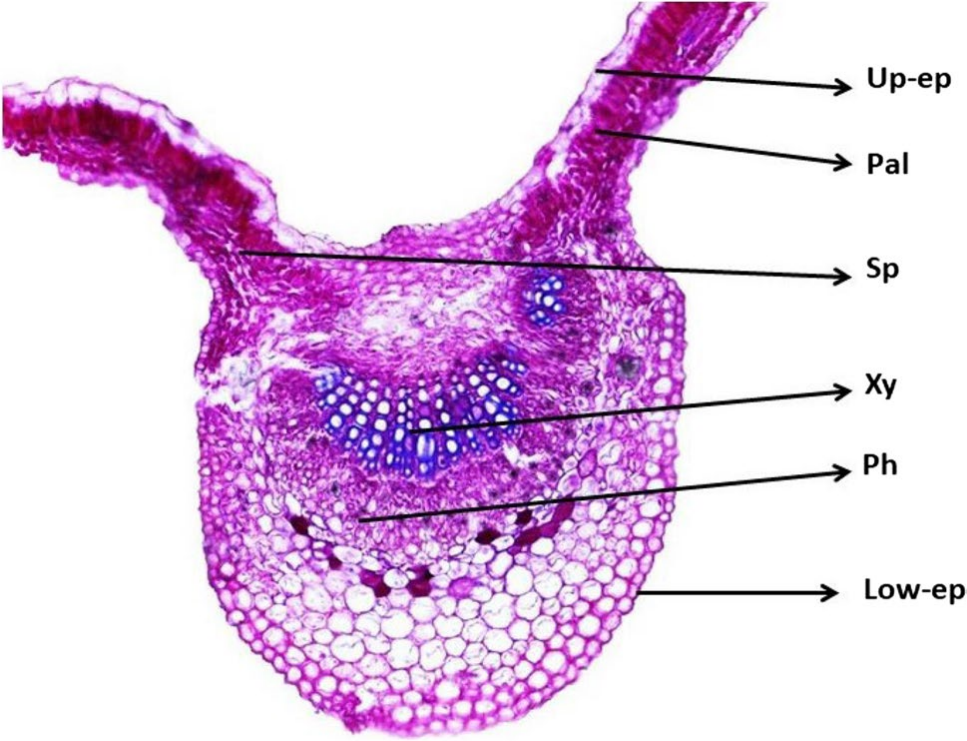
Anatomical structure of plum leaves. Up-ep: Upper epidermis, Pal: Palisade tissue, Sp: Spongy tissue, Xy: Xylem, Ph: phloem and low-ep: lower epidermis

Micro-technique procedures were carried out according to Nassar and El-Sahhar [34]. Leaf samples were collected from plants of different treatments for anatomical studies of both plum cultivars. Fourth or fifth leaf (fully expanded and mature) down from the terminal leaf was selected with total of three leaves per treatment. Leaves were killed and fixed in FAA solution using formalin, acetic acid (Merck, Darmstadt, Germany), and 70% ethyl alcohol (Chem-Lab., Zedelgem, Belgium) for at least 48 h. After fixation, the materials were washed in 50% ethyl alcohol, dehydrated through a regular butyl alcohol (SDFCL - S D Fine-Chem Ltd., Mumbai, India) series and embedded in paraffin wax (LOBA Chemie PVT. LTD., Mumbai, India). Transverse sections (five sections per sample) were cut to a thickness of 20 μm using rotary microtome (Leica RM2125 RTS, Leica Biosystems, Illinois, USA). Sections were stained with crystal violet-erythrosine before mounting in Canada balsam and fixing the cover slips. Slides were examined using microscopically (DM750, Leica Microsystems, Wetzlar, Germany) equipped with digital microscope camera (ICC50 HD, Leica Microsystems, Wetzlar, Germany) and photographed using a micrometer lens. Anatomical characteristics of leaves were measured using a Leica Light Image Analysis System and three readings per slide were averaged. The anatomical characteristics of leaves including the thicknesses of lamina, upper epidermis, palisade tissue, spongy tissue, lower epidermis, midvein, xylem and phloem were measured as described in Fig. 1.

### Experimental design and statistical analysis

The four treatments were arranged in a complete randomized block design, with three replicates per treatment. Each experimental unit (replication) consisted of 3 plum plants (*n* = 36 plants for each cultivar). Shapiro–Wilk’s test (*P* ≤ 0.05) was used to determine whether the dataset follows a normal distribution. Transformation was applied in case of data were not normally distributed for further analysis. Normalized data were used in statistical analysis, but all results are shown as original data. One-way analysis of variance (ANOVA) was performed using the general linear model (GLM) procedure—SAS software Version 9.0 (SAS Institute Inc., North Carolina, USA). Significant differences (α ≤ 0.05) were determined using Duncan’s multiple range test [35]. Results were plotted as the mean ± standard error (SE) using Microsoft Excel - office professional plus 2021 (version 16.0, Microsoft, Inc., Redmond, WA, USA).

## Results

### Transmission electron microscopy (TEM)

**Fig. 2:**
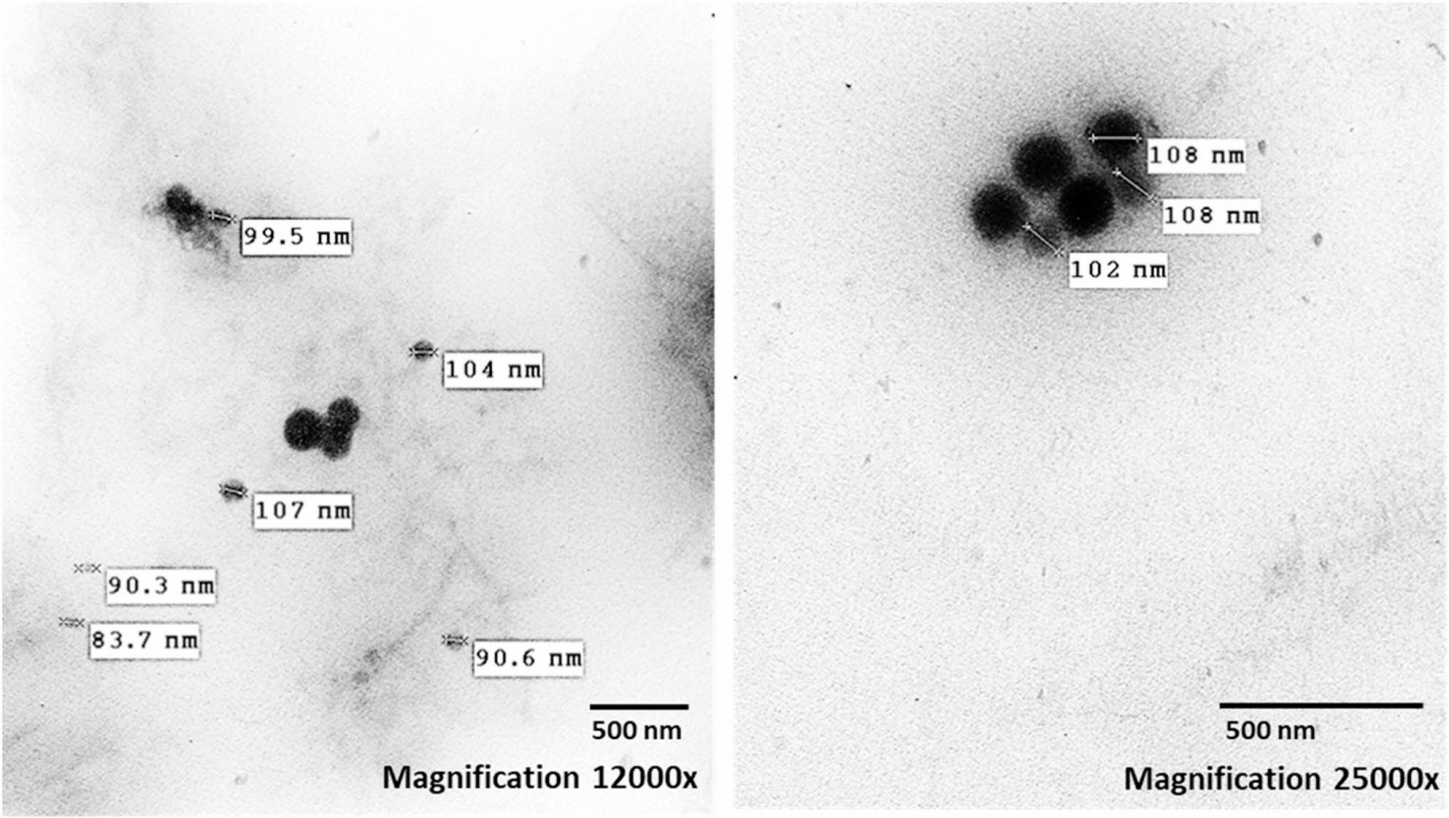
Transmission electron microscopy images of ChNPs at various magnifications

The morphology of the nanoparticles is studied using a transmission electron microscope. The TEM images revealed that ChNPs were stable with spherical shape,

dark color in core and surface, uniform size distribution and smooth surface morphology. ChNPs size was ranged 83.7–108 nm (Fig. 2).

### Dynamic light scattering (DLS) analysis

**Fig. 3:**
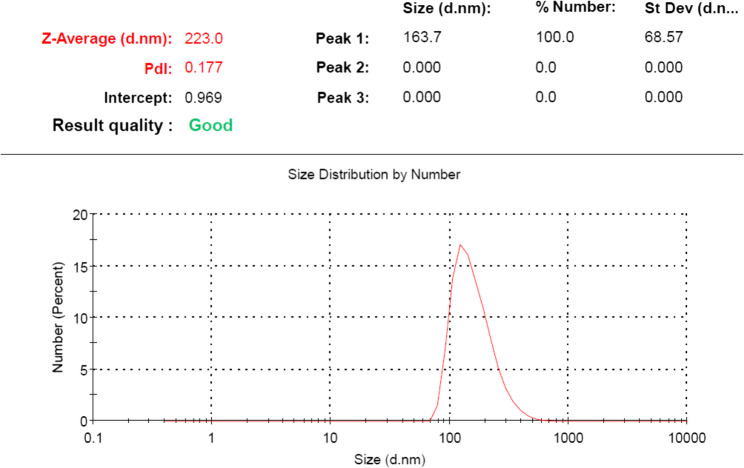
Size distribution by number of ChNPs by dynamic light scattering (DLS)

Hydrodynamic diameter was measured in the nanoscale range using the DLS technique (Fig. 3). DLS analysis showed average diameter of ChNPs about 163.7 ± 68.5 nm. PDI value was 0.177 which indicated homogenous and appropriate distribution of ChNPs.

### Plant growth parameters

**Fig. 4:**
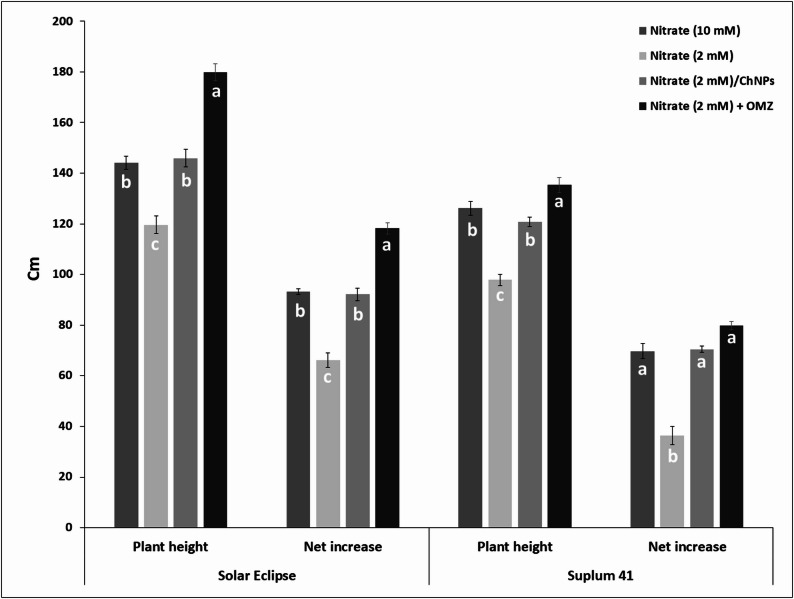
Effect of N treatments on plant height (cm) of Solar Eclipse and Suplum 41. Vertical bars represent the standard error (SE) of means. Different letters indicate significant differences according to Duncan’s test (*p* = 0.05)

**Fig. 5:**
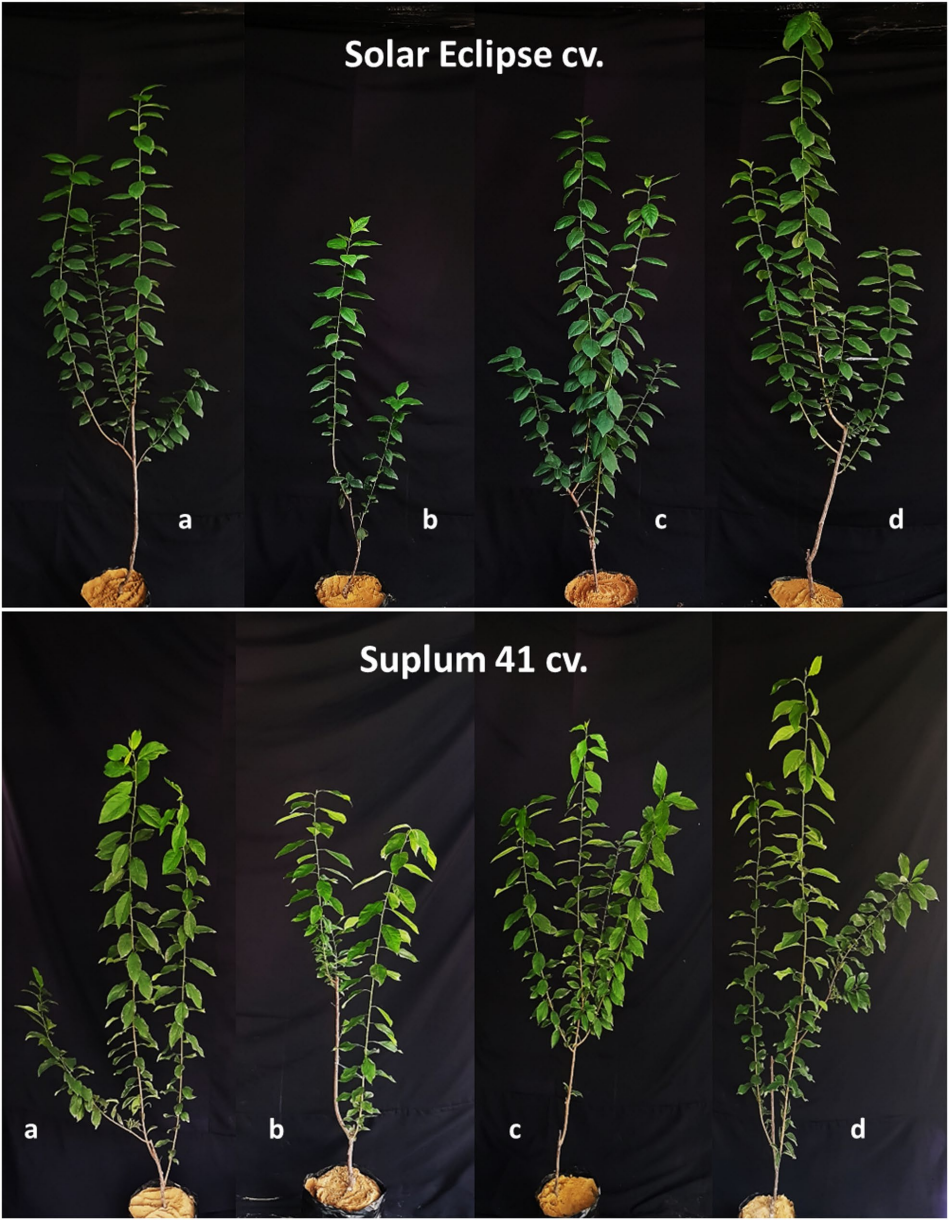
Differences in growth vigor of Solar Eclipse and Suplum 41 plants. **a)** Plants received high N (10 mM NO_3_^−^), **b)** Plants received low N (2 mM NO_3_^−^), **c)** Plants received ChNPs-NO_3_^−^ (2mM), **d)** Plants received OMZ + NO_3_^−^ (2mM)

Plant height was affected by different treatments of nitrogen in both plum cultivars (Figs. 4 and 5). The lowest value of plant height was recorded in plum plants received 2 mM NO_3_^−^ (low N) in both cultivars. The highest value of plant height was noticed in plants treated with low N combined with OMZ for Solar Eclipse with net increase 118.17 cm and for Suplum 41 with net increase 79.83 cm. Moreover, net increase of height in OMZ treated plants was significantly higher with 14.6–26.8% than plants received high N and 78.3–118.7% than plants received low N related to cultivar. Application of ChNPs-NO_3_ (2 mM) increased plant height with 21.9–23.5% higher than plum plants received 2 mM NO_3_^−^ only based on cultivar. Although, applied N rate was different, no significant differences in plant height of both cultivars were observed between plants received 10 mM NO_3_^−^ (high N) and that treated with ChNPs-NO_3_ (2 mM).

**Fig. 6:**
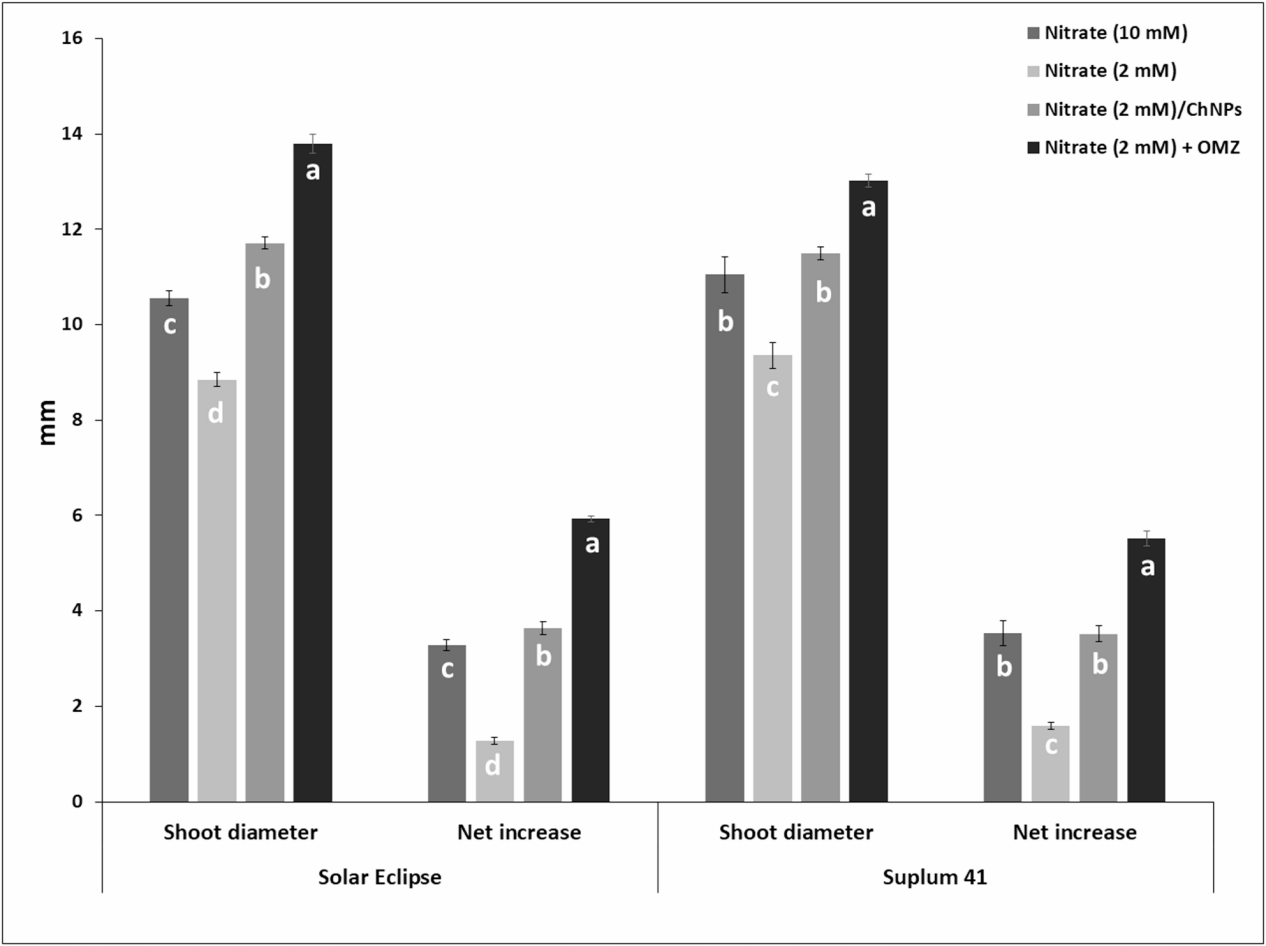
Effect of N treatments on shoot diameter (mm) of Solar Eclipse and Suplum 41. Vertical bars represent the standard error (SE) of means. Different letters indicate significant differences according to Duncan’s test (*p* = 0.05)

Addition of OMZ to low rate of nitrogen increased shoot diameter (mm) in both cultivars compared to plants received low or high N rate (Fig. 6). OMZ recorded the highest shoot diameter with net increase about 5.9 mm in Solar Eclipse and 5.5 mm in Suplum 41. Increase rate was about 39.2–55.9% compared to plants received low N rate and 17.9–30.7% compared to plants received high N rate based on cultivar. ChNPs-NO_3_ increased shoot diameter to 11.7 and 11.5 mm for Solar Eclipse and Suplum 41, respectively. Significant differences were found in shoot diameter as it increased due to application of ChNPs-NO_3_ compared to low and high N treatments in Solar Eclipse and low N in Suplum 41. The lowest value of shoot diameter was recorded in plants received low N (2 mM NO_3_^−^) with net increase only 1.28 mm in Solar Eclipse and 1.60 mm in Suplum 41.

**Fig. 7:**
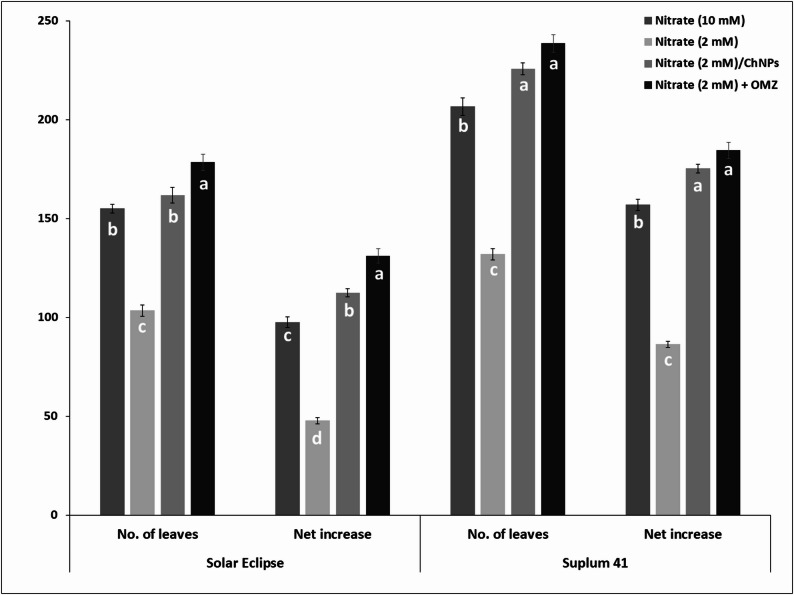
Effect of N treatments on number of leaves of Solar Eclipse and Suplum 41. Vertical bars represent the standard error (SE) of means. Different letters indicate significant differences according to Duncan’s test (*p* = 0.05)

Number of leaves is an important indicator for vegetative growth and affected by different N treatments (Fig. 7). Application of NO_3_^−^ at 2 mM combined with OMZ or loaded on ChNPs had significant effects on number of leaves. Plum plants treated with OMZ and low N produced more leaves than other treatments (Fig. 5). OMZ treated plants with low N recorded increase in number of leaves about 72.5–80.8% compared to plants received low N (2mM NO_3_^−^) based on cultivar and 15% compared to plants received high N (10mM NO_3_^−^) for both cultivars. Using ChNPs-NO_3_^−^ resulted in higher number of leaves per plant by 56.4% for Solar Eclipse plants and 71% for Suplum 41 compared to plants treated with low nitrogen only.

### Leaf characteristics

**Fig. 8:**
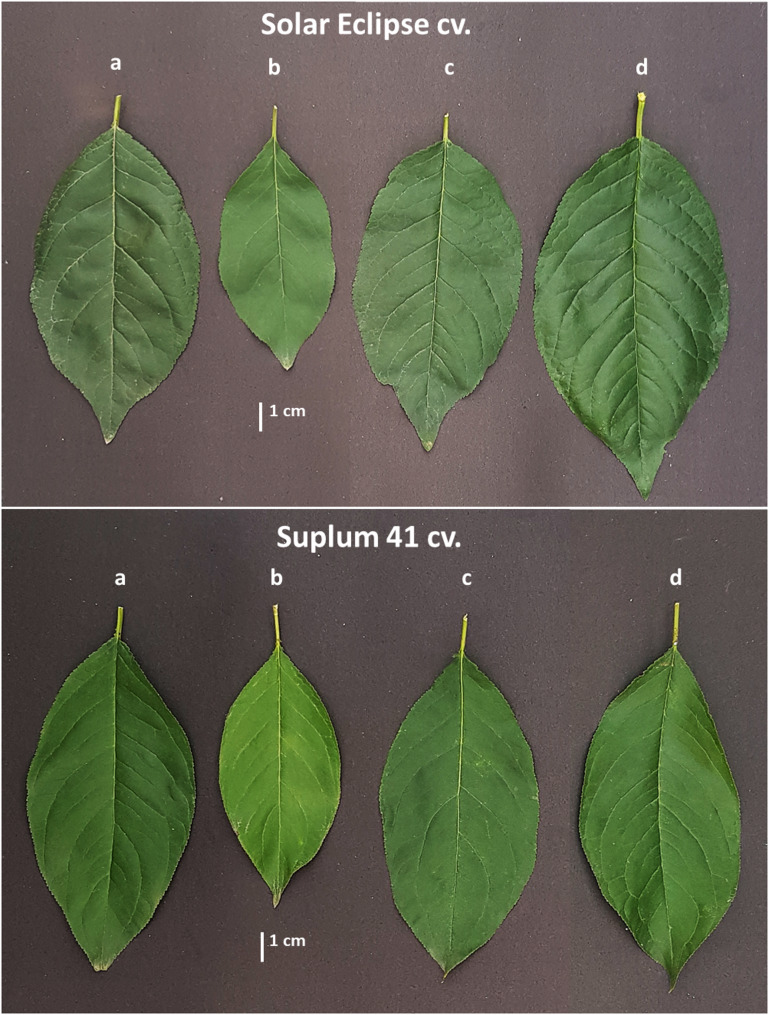
Differences in leaf area of Solar Eclipse and Suplum 41 plants. **a)** Plants received high N (10 mM NO_3_^−^), **b)** Plants received low N (2 mM NO_3_^−^), **c)** Plants received ChNPs-NO_3_^−^ (2mM), **d)** Plants received OMZ + NO_3_^−^ (2mM)

Changes in leaf characteristics of plum plants due to application of ChNPs and OMZ were observed (Table 1). Plants received low N (2 mM NO_3_^−^) combined with OMZ recorded the highest leaf area for both cultivars. Leaf area of OMZ treated plants was larger by 38.3% for Solar Eclipse and 51.4% for Suplum 41 when compared to Low N treatment (Fig. 8). Moreover, when compared to high N plants, leaf area was larger by 18.7–23.3% depend on cultivar. Using Nano form of nitrogen fertilizer had a significant effect on leaf area. ChNPs-NO_3_ increased leaf area even compared to low or high N plants. The Increase was about 27.4–35.5% compared to low N plants and 9.2–10.1% compared to high N plants based on cultivar. The lowest value of leaf area was recorded in plants of both cultivars that received 2 mM NO_3_ only.

**Table 1:** Effect of N treatments on leaf characteristics of Solar Eclipse and Suplum 41 plants

Treatment	Length (cm)	Width (cm)	Area (cm^2^)	Total leaf area (m^2^)
Solar Eclipse				
NO_3_^−^ (10mM)	9.53 b	5.22 c	34.17 c	0.53 b
NO_3_^−^ (2mM)	8.67 c	4.91 d	29.32 d	0.30 c
ChNPs-NO_3_ (2mM)	9.84 ab	5.52 b	37.34 b	0.61 b
OMZ + NO_3_^−^ (2mM)	10.13 a	5.82 a	40.55 a	0.72 a
Suplum 41				
NO_3_^−^ (10mM)	9.44 c	4.64 b	30.08 c	0.62 c
NO_3_^−^ (2mM)	8.53 d	4.17 c	24.44 d	0.32 d
ChNPs-NO_3_ (2mM)	9.98 b	4.83 b	33.11 b	0.75 b
OMZ + NO_3_^−^ (2mM)	10.57 a	5.13 a	37.00 a	0.89 a

* Different letters within each column indicate significant differences according to Duncan’s multiple-range test (*p* = 0.05)

Significant differences in leaf area were noticed due to increase in leaf dimensions (length and width) as a result of using OMZ or ChNPs. Regarding plant total leaf area, the same trend in results were observed through the significant effect of OMZ or ChNPs on number of leaves per plant and leaf area.

### Photosynthetic pigments

Leaves color of OMZ or ChNPs treated plants under low N conditions were greener compared to plants received low N only (Fig. 8). Plants received low nitrogen (2 mM NO_3_^−^) only recorded the lowest value of Chl_a_, Chl_b_ and total Chl_a+b_ in both cultivars (Table 2). Application of ChNPs-NO_3_ led to significant increase in total chlorophyll content by 16.7% in Solar Eclipse leaves and 23.1% in Suplum 41 leaves compared to plants treated with low N. Also, no significant differences were observed when compared to high N. Addition of OMZ to low dose of NO_3_^−^ resulted in highest total chlorophyll content in Solar Eclipse and Suplum 41 leaves. Increase rate was 23.6–38% in Chl_a_, 65.6–70% in Chl_b_ and 31.6–43.5% in total chlorophyll content compared to low N treatment based on cultivar. Same trend was observed in chlorophyll index (SPAD). Application of OMZ with low N increased SPAD-chlorophyll values about 19.7% for Solar Eclipse and 27.9% for Suplum 41 compared to leaves of plants received low N only (Table 2). Despite N dose was fivefold, no significant differences in SPAD-chlorophyll values and total chlorophyll were observed between plants treated with ChNPs-NO_3_ and high N treatments. Plants received low N showed the lowest SPAD-chlorophyll values in both cultivars.

**Table 2:** Effect of N treatments on leaf photosynthetic pigments of Solar Eclipse and Suplum 41 plants

Treatment	Chl_a_ (mg/g FW)	Chl_b_ (mg/g FW)	Total Chl_a+b_ (mg/g FW)	Chl index (Spad)
Solar Eclipse				
NO_3_^−^ (10mM)	3.11 ab	0.96 a	4.07 ab	51.62 b
NO_3_^−^ (2mM)	2.58 c	0.64 b	3.23 c	45.30 c
ChNPs-NO_3_ (2mM)	2.86 b	0.91 a	3.77 b	50.39 b
OMZ + NO_3_^−^ (2mM)	3.19 a	1.06 a	4.25 a	54.24a
Suplum 41				
NO_3_^−^ (10mM)	2.53 b	0.75 a	3.28 b	44.84 b
NO_3_^−^ (2mM)	2.05 c	0.50 b	2.55 c	37.31 c
ChNPs-NO_3_ (2mM)	2.41 b	0.73 a	3.14 b	43.84 b
OMZ + NO_3_^−^ (2mM)	2.83 a	0.85 a	3.66 a	47.73 a

* Different letters within each column indicate significant differences according to Duncan’s multiple-range test (*p* = 0.05)

### Total nitrogen (%)

**Fig. 9:**
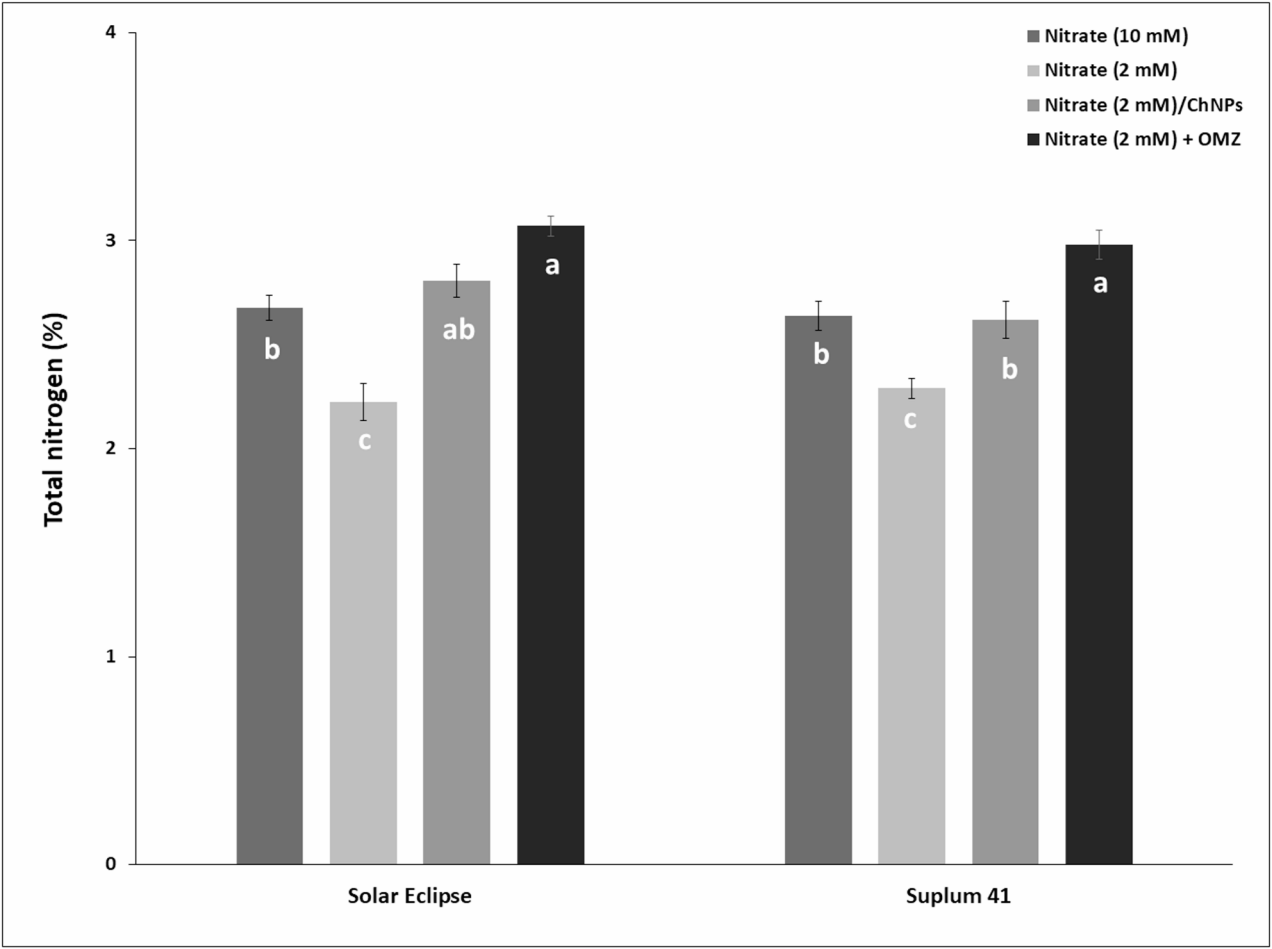
Effect of N treatments on leaf total nitrogen (%) in Solar Eclipse and Suplum 41. Vertical bars represent the standard error (SE) of means. Different letters indicate significant differences according to Duncan’s test (*p* = 0.05)

Total nitrogen (%) was determined in leaves of plum plants to verify absorption efficiency of N under experiment conditions (Fig. 9). As expected, N % was lowest in plum plants treated with low N (2 mm NO_3_^−^) only in both cultivars. Leaves of plants received low N combined with OMZ recorded highest N content with increase 30.1–38.2% compared to low N treatment and 12.8–14.6% compared to high N based on cultivar. Addition of ChNPs-NO_3_ resulted in higher N leaf content with increase 14.4–26.6% compared to low N treatment depend on cultivar. Although the nitrogen rate is 5 times higher, no significant differences were found between high N (10 mM NO_3_^−^) and ChNPs-NO_3_ (2 mm NO_3_^−^).

### Anatomical characteristics of leaves

**Fig. 10:**
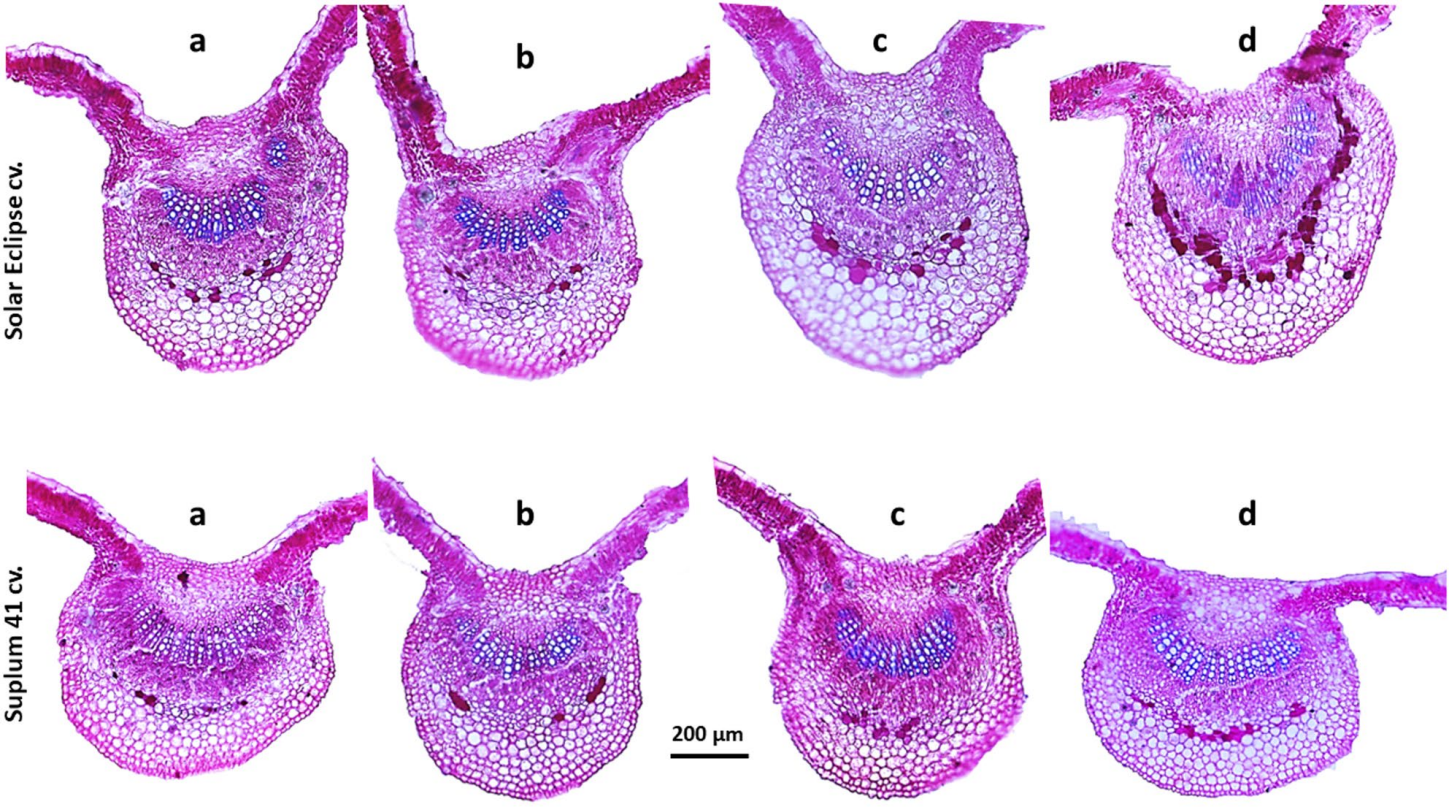
Transverse sections through lamina of the leaf developed at the median portion of the main stem showing effect of different N treatments on leaves anatomical characters of plum cultivars. **a)** Plants received high N (10 mM NO_3_^−^), **b)** Plants received low N (2 mM NO_3_^−^), **c)** Plants received ChNPs-NO_3_^−^ (2mM), **d)** Plants received OMZ + NO_3_^−^ (2mM)

Data presented in Table (3) showed that the tested approaches improved leaf anatomical characteristics compared to the other treatments in both cultivars (Table 3 - Fig. 10). Low N (2mM NO_3_^−^) treatment recorded the lowest values in all anatomical features in both cultivars. Most of anatomical characteristics were improved by application of OMZ as recorded the highest values compared to plants received low or high N only. In OMZ treated plants, increase rate was about 11.2–69.5% in the lamina, 32.8–36.7% in the upper epidermis, 9.1–59.9% in the palisade tissue, 13.1–79.9% in the spongy tissue, 33–47.3% in the lower epidermis, 39.2–61.9% in the xylem and 21.2–54.1% in the phloem in comparison with plants received low N (2 mM NO_3_^−^) according to plum cultivar. Effect of ChNPs-N fertilizer on leaf anatomical characteristics followed a comparable pattern to OMZ treatment. Increase rates were about 16.3–63.5% in the Lamina, 19.1–24% in upper epidermis, 19.9–53.7% in the palisade tissue, 17.4–63.6% in spongy tissue, 6.8–31.9% in the lower epidermis, 17.4–33.5% in the xylem and 11.9–34.6% in phloem when compared to plants received low N only related to plum cultivar. The highest value of lamina thickness was observed by application of OMZ in Solar Eclipse leaves, while it was achieved by using of ChNPs in Suplum 41 leaves. In addition, increase rate of palisade tissue thickness by ChNPs was higher than OMZ in Suplum 41 leaves, while no significant differences were observed between ChNPs and OMZ treatments in Solar Eclipse leaves. Furthermore, significant increase in thickness of leaf midvein by application of ChNPs was recorded higher than OMZ and high N treatments in both cultivars. It is noteworthy that OMZ had significant impact higher than ChNPs with increase rate of 13.6–15.4% in xylem size and 10.4–26.7% in phloem size compared to high N (10 mM NO_3_^−^) based on plum cultivar.

**Table 3:** Effect of N treatments on leaves anatomical structure of plum cvs. Solar Eclipse and Suplum 41

**Cultivar**	**Treatment**	**Thickness of anatomical characters (μm)**							
		**Lamina**	**Upper epidermis**	**Palisade tissue**	**Spongy tissue**	**Lower epidermis**	**Midvein**	**Xylem**	**Phloem**
Solar Eclipse	NO_3_^-^ (10 mM)	97.0 c	22.9 b	44.4 b	49.6 c	19.1 c	661.8 c	127.9 b	92.7 b
	NO_3_^-^ (2mM)	68.8 d	19.8 c	34.8 c	33.4 d	16.3 d	611.9 d	91.2 c	84.4 bc
	ChNPs-NO_3_ (2mM)	112.5 b	23.6 b	53.4 a	55.0 b	21.5 b	766.9 a	121.7 b	94.4 b
	OMZ + NO_3_^-^ (2mM)	116.6 a	26.3 a	55.6 a	60.1 a	24.0 a	717.3 b	147.6 a	102.3 a
Suplum 41	NO_3_^-^ (10 mM)	93.0 c	22.6 c	43.8 c	47.0 ab	21.0 a	568.2 c	105.3 b	82.5 c
	NO_3_^-^ (2mM)	87.9 d	19.5 d	40.7 d	42.8 b	17.1 b	578.8 bc	85.9 c	67.8 d
	ChNPs-NO_3_ (2mM)	102.2 a	24.2 b	48.8 a	50.3 a	18.3 b	611.3 a	100.8 b	91.3 b
	OMZ + NO_3_^-^ (2mM)	97.7 b	26.7 a	46.3 b	48.5 a	22.8 a	588.5 b	119.6 a	104.5 a

* Different letters within each column indicate significant differences according to Duncan’s multiple-range test (*p* = 0.05)

## Discussion

### Characterization of ChNPs

ChNPs were characterized concerning size and morphology in addition to shape and surface by TEM and DLS analyses. TEM images of this study revealed that the ChNPs are often spherical shaped with dark colored and diameter ranged of 83.7–108 nm. Similarly, it was found that diameter of ChNPs was less than 100 nm as shown in TEM images [28]. Furthermore, same nanoparticles morphology was recorded as ChNPs were nearly spherical shaped with an amorphous nature and smooth external surface but with higher size range of 150–350 nm [36]. Dark color of ChNPs is referred to regions with higher electron density distribution, so it can be concluded that chitosan has high level of cross-linking density in these regions with TPP [37]. In the present study, the results of DLS analysis showed average diameter of ChNPs about 163.7 ± 68.5 nm with PDI value was 0.177 which indicated homogenous and appropriate distribution of ChNPs. Similar results found that the size distribution profile by DLS analysis had average diameter of ChNPs about 192.2 ± 2.5 nm [38]. Recently, DLS analysis of ChNPs prepared with ionic gelation method showed similar particles size with an average of 172.8 ± 3.9 nm with PDI value of 0.166 [39]. Further, PDI value ˃ 0.5 represents polydispersity (heterogeneous) and PDI value ˂ 0.5 normally shows the monodispersity (homogeneous). Generally, PDI value of monodisperse ChNPs is within range of 0.2–0.4 [40]. The variations in ChNPs size between DLS and TEM (slightly larger by DLS) could be explained as DLS measures a hydrodynamic diameter in hydrated state, while TEM measures an actual diameter in dry state [37].

### Growth behavior

Application of OMZ on fruit trees has remained unexplored until now. This is the first report that clarifying effect of OMZ on plum plants as a model of fruit trees. Application of OMZ under low N conditions enhanced growth parameters compared to plum plants received low or high N. The obtained results appear to confirm previous report which revealed that addition of OMZ with low N in the growth media had a significant impact by enhancing plant growth that declined because of N stress. Leaf area in OMZ treated plants was 61% larger than control plants under low N conditions [19]. OMZ application increased growth parameters, including plant height, number of leaves and leaf area compared with the control in peppermint plants [26]. Leaf area was expanded in response to OMZ treatment in basil plants. Additionally, basil cultivars showed different response to OMZ treatment related to height and stem diameter [24]. Plant growth improved by OMZ application could be connected to increase of nutrient use efficiency as a result of the root system stimulation [22]. It has been proved that OMZ absorption via root systems improves plant growth as it characterized with signalling and/or hormonal action [24].

Many researchers investigated the essential role of ChNPs as plant growth promoters [41, 42]. Results of the current research clarified that application of ChNPs with low N dose significantly increased growth parameters including plant height, stem diameter, number of leaves and Leaf area. Similarly, ChNPs improved the vegetative growth compared to control and chitosan treated seedlings of coffee [43]. Also, ChNPs treatment promoted growth parameters of pepper seedlings [44]. Furthermore, similar results showed the potential of ChNPs-NPK as growth enhancer of wheat plants [45]. Using ChNPs-NPK significantly increased all growth parameters of coffee seedlings after 60 days of treatment. Plant height was increased by 12.7%, stem diameter by 28.3%, number of leaves by 22.8% and leaf area by 46.8% compared to control [29]. ChNPs augmented leaf area of wheat plants compared to the control [46]. Moreover, leaf area of maize plants exhibited notable increasement after treatment with ChNPs [47].

### Leaf chlorophyll content

Photosynthetic pigments have a pivotal role in photosynthesis including absorption, transmission, and transformation processes of light energy [48]. N fertilization has a significant impact on regulation of photosynthetic pigment synthesis in plant leaves [49, 50]. Moreover, a positive correlation was recorded between N-level supply and chlorophyll content [51, 52]. SPAD values could be a good indicator of photosynthetic process through measuring leaf chlorophyll content which is important for monitoring plant physiological status [53]. In this study, leaves color of OMZ or ChNPs treated plants under low N conditions were greener compared to plants received low N only. The data obtained agree with those reported by Elansary and El-Abedin [26] who indicated that application of OMZ led to increase in chlorophyll content which combined with high photosynthetic activity. Similarly, significant increase in SPAD values of basil leaves after the application of OMZ under greenhouse conditions [23]. Also, leaves of plants treated with OMZ were also visibly greener and SPAD values were fivefold higher than untreated plants under low N conditions [19]. OMZ could delay leaves senescence state by adequate NO_3_^−^ uptake [54] that leading to maintain integrity of chloroplasts and high photosynthetic activity [55]. Moreover, high photosynthetic activity induced by OMZ may be linked to increase expression of the key photosynthetic genes [21, 54]. In addition to this, ChNPs also increased leaf chlorophyll content, which synergistically increase leaf photosynthesis activity [46, 56]. Nano-fertilizer based on ChNPs enhanced chlorophyll content in the coffee leaves compared to the control with an increase 14.8–27.7% in chlorophyll a and 14.8–27.7% in chlorophyll b [29]. It has been suggested that increase in leaf chlorophyll content may be attributed to inducing chloroplast gene expression by chitosan [57, 58].

### N uptake efficiency

Addition of OMZ and ChNPs-NO_3_ increased leaf N content compared to control under low N conditions based on cultivar. OMZ application dose influenced NO_3_^−^ uptake by root system from the soil. At low NO_3_^−^ concentrations, NO_3_^−^ uptake was increased by 30% at 1 µM OMZ and 27% at 10 µM OMZ [21]. Previous studies showed an increase in leaf NO_3_^−^ content due as a result of OMZ treatment in tomato [21, 22] and basil [23, 24]. OMZ application may induce ability of nutrient acquisition from the soil via improving nutrient uptake and translocation [22]. It has been hypothesized that OMZ may enhance NUE through N uptake and assimilation and regulate several metabolic pathways that reflected on enhancing plant growth [19]. ChNPs may induce many biological responses in the plants which related directly or indirectly with an increase in nutrients uptake in plant leaves [59]. ChNPs improve nutrient uptake with slow-release feature which can decrease frequency of nutrients addition to the soil by supplying plants with required nutrients over an extended time [17, 60]. Using ChNPs increased Nitrogen uptake by 9.8–27.4% [43]. Application of ChNPs-NPK at different rates increased N uptake by 17.04% compared to control in coffee seedling [29]. Using ChNPs-NPK in fertilization of wheat and rice plants led to lower N loss and high N uptake efficiency through addition of a slow-release feature to the nano composite [45, 61]. ChNPs exhibited slow release of N nutrient (NO_3_^−^) with relative release about 13% for more than 48 h due to force of the ionic bond between ChNPs in positive charge and N (NO_3_^−^) in negative charge [29]. It was found that application of nano nitrogen fertilizer reduced NO_3_^−^ leaching [62]. Chitosan could enhance the anion exchange capacity of the soil due to high affinity towards anionic nutrients such as nitrate (NO_3_^−^) because of its cationic nature, so it can reduce leaching of NO_3_^−^ [63, 64].

### Changes in leaf anatomical structure

This is the first report that illustrates the effect of OMZ on leaf anatomical structure. Plant growth and metabolism is affected by changes in leaf anatomical structure which is the basis of physiological functions in leaves [65]. Leaves are mainly composed of an epidermis, mesophyll tissue, and a vascular system [66]. Mesophyll comprised 45–54%, epidermis 20–28%, and vascular bundles 6–9% of transverse leaf area depend on genotype and N treatment [67]. Previous studies had revealed that N addition increased the xylem vessels size and its permeability efficiency in leaf veins which resulted in reduction of leaves construction cost [68–70]. Under low N conditions in this experiment, OMZ and ChNPs generally enhanced leaf anatomical structure of both cultivars. Application of ChNPs led to progressive increasing in thickness of many anatomical parts of leaves including epidermal cells, palisade tissue, spongy tissue, number of xylem arms and phloem layers compared to leaves of untreated potato plants [71]. Moreover, ChNPs increased the thickness of blade, lower and upper epidermis, mesophyll tissue and vascular bundles compared to the control in wheat leaves [72]. The increase in the mesophyll thickness because of N fertilization application may be associated with high number of chloroplasts found in the mesophyll cells [73] and coupled with an increase in chlorophyll content which is connected to an enhanced photosynthetic activity [74]. It seems that maximizing vegetative growth in plum plants under low N conditions has relation with changes in leaf anatomical structure. Results in this study revealed that OMZ and ChNps increased the size of: (1) palisade tissue with high density of chloroplasts which is crucial for light absorption and photosynthesis efficiency [75], (2) xylem that enhance transport capacity of water and nutrient at a higher rate to leaves [76, 77], and (3) phloem which facilitates translocation of photosynthates produced during photosynthesis from the leaves to other parts of the plant [77].

## Conclusion

It has become imperative to enhance growth and maintain healthy status of plants with low utilization of chemical fertilizers. Many approaches had been tested to increase NUE and reduce reliance on N chemical fertilizers. This study is the first report about application of OMZ on fruit trees (plum) with anatomical studies of changes in leaf structure. Based on the results, Application of OMZ and ChNps has showed their potential in enhancing vegetative growth and leaf anatomical structure (xylem and phloem) of plum under low N conditions. These results support utilizing OMZ and ChNPs among next-generation biostimulant products and fertilizers. However, further studies should be conducted on fruits trees in productivity stage to verify the competence of these compounds at field trials level.

## Data Availability

All of the data supporting the findings of this study are included within the article. The datasets analyzed in the current study are available from the corresponding author upon reasonable request.

## Abbreviations

OMZ: Omeprazole
ChNPs: Chitosan nanoparticles
NUE: Nitrogen use efficiency
ChNPs-NO_3_: Chitosan nanoparticles loaded with nitrate
TEM: Transmission Electron Microscopy
Ch: Chitosan
STPP: Sodium Tripolyphosphate
PDI: Polydispersity index
DLS: Dynamic light scattering
Chl_a_: Chlorophyll A
Chl_b_: Chlorophyll B
FAA: Formalin-Acetic Acid-Alcohol solution
